# Prevalence of Malaria Infection in Pregnant Women Attending Antenatal Clinics in Southern Senegal

**DOI:** 10.4269/ajtmh.23-0164

**Published:** 2024-01-02

**Authors:** Marie Pierre Diouf, Safietou Kande, Mary Aigbiremo Oboh, Isaac Akhénaton Manga, Fassiatou Tairou, Amadou Seck, Abdoulaye Diallo, Aminata Colle Lo, Doudou Sow, Khadime Sylla, Magatte Ndiaye, Roger Clément Tine, Babacar Faye, Corinne Merle, Alfred Amambua-Ngwa, Paul Miligan, Jean-Louis Abdourahim Ndiaye

**Affiliations:** ^1^ Service Parasitologie Mycologie, UFR Santé Thiès, Senegal;; ^2^ Service Parasitologie Mycologie, Cheikh Anta Diop University, Dakar, Senegal;; ^3^ Rochester Institute of Technology, Rochester, New York;; ^4^ Gaston Berger University, Saint Louis, Senegal;; ^5^ World Health Organization Tropical Disease Research, Geneva, Switzerland;; ^6^ Medical Research Council Unit, London School of Hygiene & Tropical Medicine, Serekunda, The Gambia;; ^7^ London School of Hygiene & Tropical Medicine, London, United Kingdom

## Abstract

Despite marked progress in Senegal, three regions in the southeast part continue to have a high burden of malaria, but there have been no recent studies assessing the prevalence of malaria associated with pregnancy. This study aimed to determine the prevalence of malaria infection in pregnant women attending antenatal clinics in Senegal. During the malaria transmission season of 2019, pregnant women attending 11 health care facilities for a scheduled visit and those presenting unwell with signs of malaria were invited to participate in a malaria screening study. A finger prick blood sample was taken for malaria diagnosis by rapid diagnosis test (RDT) and polymerase chain reaction (PCR). A total of 877 pregnant women were enrolled, 787 for a scheduled antenatal consultation and 90 for an unscheduled consultation with signs of malaria. The prevalence of *Plasmodium falciparum* among the first group was 48% by PCR and 20% by RDT, and that among the second group was 86% by PCR and 83% by RDT. RDT sensitivity in capturing asymptomatic, PCR-positive infections was 9.2% but ranged from 83% to 94% among febrile women. The prevalence of infection by PCR in women who reported having received at least three doses of sulfadoxine pyrimethamine (SP) was 41.9% compared with 58.9% in women who reported they had not received any SP doses (prevalence ratio adjusted for gravidity and gestational age, 0.54; 95% CI, 0.41–0.73). The burden of *P. falciparum* infections remains high among pregnant women, the majority of which are not captured by RDT. More effective measures to prevent malaria infection in pregnancy are needed.

## INTRODUCTION

Senegal has made considerable progress in the fight against malaria and remains one of the leaders in the testing and large-scale application of new recommendations and innovative strategies.^1^ These different strategies have led to significant progress in the fight against malaria. However, malaria remains a public health problem in Senegal, with an uneven distribution across the different regions. Of the total malaria confirmed cases in 2021, 421,471 cases were registered in the Kedougou, Kolda, and Tambacounda regions, representing 78.5% of the total.^1^ These three regions also registered 46.3% of malaria-related deaths. Now, Senegal faces a changing epidemiological profile and should propose targeted strategies. The National Malaria Control Programme (NMCP) is currently focusing on improving malaria control interventions in high-incidence areas (Kedougou, Kolda, and Tambacounda zone) and on elimination activities in areas of very low incidence (in the north of the country).^2^ In addition to interventions targeted to specific zones, other specific control strategies have been implemented for vulnerable groups, including pregnant women. Protecting pregnant women is a major component of our Senegal health policy, due to the risks to both the woman and her unborn child. Malaria in pregnancy leads to serious adverse effects for the mother and the fetus.^3^ Although malaria in pregnancy might be asymptomatic due to the high level of acquired immunity in multigravida mothers residing in high-transmission areas, it can be associated with maternal anemia, abortion, preterm birth, and low birth weight.^4^ Recurrent infections with placenta strain-specific *Plasmodium falciparum* is common among women in areas of high endemicity during their first and second pregnancies.^5^^,^^6^ Prevention and control strategies are used to combat the negative consequences of malaria for women and their fetuses. These include insecticide-treated mosquito nets, intermittent preventive treatment of pregnant women (IPTp), and effective treatment of confirmed cases of malaria with artemisinin-based combination therapies (ACTs).

It is therefore important to evaluate the contribution of these different strategies over time I order to adjust them. The NMCP’s routine surveillance system enables us to monitor the evolution of malaria cases. However, it is important to carry out specific studies in pregnant women to assess the burden of malaria. These studies accompany the implementation of the strategic plan set up by the NMCP and help answer specific questions linked to the execution of activities. However, to our knowledge, no specific study to determine the prevalence of malaria in pregnant woman has been carried out in Senegal for several years. In 2008, a study was carried out in Dakar to assess the production of immunoglobulins directed against the MSP1, GLURP, and DBL5 proteins in pregnant women receiving sulfadoxine pyrimethamine (SP) for IPT.^7^ Between 2013 and 2014, a nationally representative data set was used to investigate factors influencing optimal use of IPTp and insecticide-treated nets among women with a recent pregnancy in Senegal.^8^ To address this gap, we conducted this study in low- and high-transmission areas in Senegal to determine the prevalence of malaria infection and its associated risk factors among pregnant women attending antenatal consultation (ANC) at health facilities.

## MATERIALS AND METHODS

### Study site and population.

**Figure 1.**
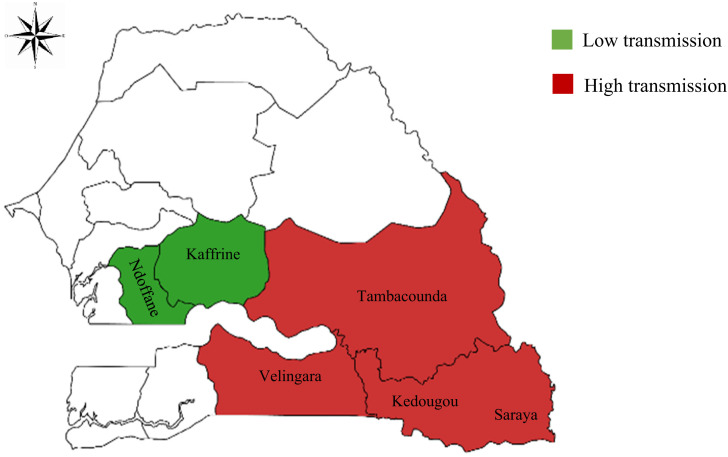
Study site (MapChart: https://www.mapchart.net/africa-detailed.html; personal data).

A cross-sectional study was conducted between September 2019 and January 2020. The study collected data and samples from pregnant women attending one of 11 health facilities. Health posts were located within six districts (Figure 1) of central and southern Senegal, four of which (Tambacounda, Velingara, Kedougou, and Saraya) were in zones of high endemicity (malaria incidence of ≥ 25 cases/1,000 population) and two of which (Kaffrine and Ndoffane) were in zones of low endemicity (malaria incidence of 5–15 cases/10,000 population).^9^ Health posts were selected based on the geographical accessibility during the rainy season and the willingness of health facility staff to participate in the study. In the study area, malaria transmission is highly seasonal, with peak transmission occurring between October and November. *Plasmodium falciparum* is the predominant parasite species circulating in the area, and transmission is due mainly to *Anopheles gambiae* s.l.^10^ In these districts, reported coverage of IPTp is higher in low-transmission areas than in high-transmission areas; the proportion of women who reported receiving three doses of IPTp ranged from 75.8% to 83.6% across low-transmission districts compared to 44.2–61.7% in high-transmission districts.^11^

For each health facility, women who reported residing in the study district for the previous 6 months and were attending a scheduled or unscheduled antenatal care visit were eligible to participate in the study. Women who reported a known allergy to SP or were HIV positive were excluded from participating.

### Data collection.

After written informed consent was obtained, a structured questionnaire was administered to collect information about age, residence, gravidity, and use of long-lasting insecticidal nets (LLINs) and IPTp with SP (IPTp-SP). Blood samples were taken by finger prick to check for malaria parasites by using a *P. falciparum* histidine-rich protein 2 malaria rapid diagnosis test (RDT) (SD Bioline^TM^; Standard Diagnostics, Giheung-ku, Republic of Korea). Three dried blood spots per participant were collected on Whatman filter paper for molecular analysis. Women who had a positive RDT were treated with ACT according to the national recommendations. Women attending a routine visit who had a negative RDT were given SP, except for those who were in the first trimester. Women attending an unscheduled consultation who had a negative RDT were not treated with SP until their next scheduled appointment.

### Laboratory methods.

DNA was extracted from dried blood spot samples using the Qiagen (QIAamp^®^) protocol (manufacturer’s instructions were followed). DNA was eluted in 100 µL and kept in a –20°C freezer until sample processing. Parasitemia was confirmed by a real-time polymerase chain reaction (PCR) assay using the *var*ATS gene^12^ and the primer probe sequence and cycling conditions reported elsewhere.^13^ Briefly, 5 μL of template genomic DNA was added to a reaction mixture. This mixture contained 1 μL of nuclease-free water, 10 μL of 2× Taqman Universal PCR Mastermix (Applied Biosystems, Sparta, NJ), 1.6 μL each of forward and reverse primer (primer concentration, 10 μM), and 0.8 μL of probe (probe concentration, 10 μM). The reaction mix was run on a CFX96 real-time system thermocycler (BioRad Laboratories, Hercules, CA). For all runs, 3D7 culture isolates and DNA-free water were used as positive and negative controls, respectively.

### Statistical methods.

Data were entered in Excel 2013 and analyzed using STATA 16 (STATA Corporation, College Station, TX). Age was categorized in the age groups of 14–20, 21–25, 26–31, and 32–42 years of age, and parity was categorized as primigravidae (first pregnancy), secondigravidae (second pregnancy), multigravidae (third, fourth, or fifth pregnancy), and grande multigravidae (sixth pregnancy or more). The prevalences of infection determined by PCR were compared according to the number of IPTp-SP doses received and the use of LLINs, adjusted for district, age group, gravidity, gestational age, and month of recruitment, using Poisson regression with a robust standard error to estimate prevalence ratios.^14^^,^^15^

## RESULTS

Between September 2019 and January 2020, 879 women were invited to participate in the study. Of these, 2 refused and 877 were interviewed and provided a finger prick blood sample. The mean age was 24.4 ± 6.2 years, and 10% (90/877) had attended an unscheduled visit. Overall, 95% of women (833/877) reported having used a bed net (the previous night) and 64% (562/877) had taken at least one dose of SP (Table 1).

**Table 1:** Characteristics of the study participants (*N* = 877)

Variable	*n*	% (95% CI)
Mean age ± SD	24.4 ± 6.2	–
Age group (years)		
14–20	336	38.3 (35.1–41.6)
21–25	212	24.2 (21.4–27.2)
26–31	218	24.9 (22.1–27.9)
32–42	111	12.6 (10.6–15.1)
Gravidity		
Primigravidae	199	22.7 (20.0–25.6)
Secondigravidae	170	19.4 (16.8–22.2)
Multigravidae	414	47.2 (43.9–50.6)
Grande multigravidae	94	10.7 (8.8–13.0)
Gestational age		
1st trimester	220	25.1 (22.3–28.1)
2nd trimester	367	41.8 (38.6–45.2)
3rd trimester	290	33.1 (30.0–36.3)
Education level		
Primary	214	71.6 (61.6–82.1)
Secondary	61	20.4 (16.0–25.4)
University	3	1.0 (0.2–2.9)
Nonformal education	21	7.0 (4.0–12.1)
Marital status		
Single	13	1.5 (0.8–2.6)
Married	863	98.4 (48.3–59.9)
Divorced	1	0.1 (97.3–99.1)
ITN use	833	95.0 (93.5–96.5)
IPTp received	562	64.1 (60.8–67.2)
No. of doses		
1	263	46.8 (42.6–51.0)
2	173	30.8 (27.0–34.8)
3	86	15.3 (12.5–18.6)
4	33	5.9 (4.1–8.2)
5	7	1.2 (0.5–2.7)
ANC	787	89.7 (87.5–91.6)
Unscheduled	90	10.3 (8.4–12.5)

ANC = antenatal consultation, IPTp = intermittent preventive treatment of pregnant women, ITNs = insecticide-treated mosquito nets.

### *Plasmodium falciparum* prevalence by PCR and RDT.

Among women attending a scheduled visit (*N* = 787), 20% were positive for *P. falciparum* by RDT and 48% were positive by PCR (Table 2). Of 489/787 women who were apparently well with no reported illness symptoms, 37% (184/489) were positive for *P. falciparum* by PCR but only 4.7% (23/489) were positive by RDT. Of 124/787 women with fever or history of fever, 81% (100/124) were positive by PCR and 71% (88/124) were positive by RDT.

**Table 2:** Percentage of women testing positive by PCR and RDT

Parameter	*n*	PCR+	RDT+	Se	Sp	PPV	NPV
Scheduled visit	787	48.4% (381/787)	20.2% (159/787)	38.1% (145/381)	96.6% (392/406)	91.2% (145/159)	62.4% (392/628)
Fever	124	80.6% (100/124)	71.0% (88/124)	83.0% (83/100)	79.2% (19/24)	94.3% (83/88)	52.8% (19/36)
Other malaria symptoms	174	55.7% (97/174)	27.6% (48/174)	46.4% (45/97)	96.1% (74/77)	93.8% (45/48)	58.7% (74/126)
No malaria symptoms	489	36.6% (184/489)	4.7% (23/489)	9.2% (17/184)	98.0% (299/305)	73.9% (17/23)	64.2% (299/466)
Nonscheduled visit	90	86.7% (78/90)	83.3% (75/90)	93.6% (73/78)	83.3% (10/12)	97.3% (73/75)	66.7% (10/15)
Fever	57	86.0% (49/57)	87.7% (50/57)	98.0% (48/49)	75.0% (6/8)	96.0% (48/50)	85.7% (6/7)
Other malaria symptoms	33	87.9% (29/33)	75.8% (25/33)	86.2% (25/29)	100.0% (4/4)	100.0% (25/25)	50.0% (4/8)

+ = positive; NPV = negative predictive value of RDT; PCR = polymerase chain reaction; PPV = positive predictive value of RDT; RDT = rapid diagnostic test; Se = sensitivity of RDT; Sp = specificity of RDT.

Among women attending an unscheduled visit (*N* = 90), 63% (57/90) had fever and 37% (33/90) had other symptoms indicative of malaria. Of those who were febrile, 88% (50/57) were positive by RDT and 86% (49/57) were positive by PCR. Overall, asymptomatic PCR-positive infections were often not detected by RDT (sensitivity, 9.2%), but symptomatic infections were detected with a sensitivity of 98% (unscheduled visits with fever) and 83% (scheduled visits with fever) (Table 2).

### Risk factors for PCR-detectable parasitemia.

Prevalence among those attending a scheduled visit varied by district, ranging from 18% in Kaffrine/Ndoffane to 79% (Tambacounda). Prevalence was highest in the 21- to 25-year-old age group (54.6%) and lowest in age groups older than 25 years. Prevalence decreased slightly with increasing gravidity, but this trend was not statistically significant. Prevalence was higher in women attending in the first trimester (52.4%) than in the second and third trimester. The prevalence was 58.9% (156/265) among those who had not taken SP for prevention, 43.1% (103/239) among those with one dose, 44.0% (70/159) among those with two doses, and 41.9% (52/124) among those with three or more doses (Table 3).

**Table 3:** Association of LLIN use and receipt of IPTp-SP with infection detected by PCR: crude and adjusted prevalence ratios among women attending a scheduled antenatal visit

Variable	% (*n*)	% Prevalence by PCR (*n*)	Prevalence ratio (95% CI)		*P* value
			Crude	Adjusted	
District					
Kaffrine/Ndoffane	27.2 (214)	18.2 (39/214)	1	1	< 0.0001
Kedougou	13.1 (103)	64.1 (66/103)	3.52 (2.56, 4.84)	3.54 (2.55, 4.90)	
Saraya	23.8 (187)	44.9 (84/187)	2.46 (1.78, 3.41)	2.30 (1.66, 3.19)	
Tambacounda	12.2 (96)	79.2 (76/96)	4.34 (3.21, 5.88)	3.84 (2.80, 5.26)	
Velingara	23.8 (187)	62.0 (116/187)	3.40 (2.51, 4.62)	3.23 (2.37, 4.41)	
Age group (years)					
14–20	35.5 (279)	51.6 (144/279)	1	1	0.0489
21–25	23.3 (183)	54.6 (100/183)	1.06 (0.9, 1.3)	1.01 (0.85, 1.21)	
26–31	27.1 (213)	42.7 (91/213)	0.83 (0.68, 1.00)	0.78 (0.62, 0.97)	
32–42	14.2 (112)	41.1 (46/112)	0.80 (0.62, 1.02)	0.81 (0.60, 1.10)	
Gravidity					
Primigravidae	21.6 (170)	51.8 (88/170)	1	1	0.6515
Secondigravidae	19.6 (154)	48.7 (75/154)	0.94 (0.76, 1.17)	0.98 (0.80, 1.21)	
Multigravidae (3 to 6 pregnancies)	41.6 (327)	48.6 (159/327)	0.94 (0.78, 1.13)	1.10 (0.90, 1.35)	
Grande multigravidae (7 or more pregnancies)	17.3 (136)	43.4 (59/136)	0.84 (0.66, 1.07)	1.14 (0.84, 1.54)	
Gestational age					
1st trimester	24.3 (191)	52.4 (100/191)	1	1	0.0024
2nd trimester	41.9 (330)	47.0 (155/330)	0.90 (0.75, 1.07)	1.24 (1.04, 1.46)	
3rd trimester	33.8 (266)	47.4 (126/266)	0.90 (0.75, 1.09)	1.52 (1.20, 1.93)	
Month of recruitment in 2019					
September	28.9 (227)	38.8 (88/227)	1	1	0.9592
October	27.4 (215)	56.7 (122/215)	1.46 (1.20, 1.79)	1.06 (0.88, 1.27)	
November	29.0 (228)	49.6 (113/228)	1.28 (1.04, 1.58)	1.00 (0.82, 1.21)	
December	11.3 (89)	49.4 (44/89)	1.28 (0.98, 1.66)	1.03 (0.81, 1.32)	
January	3.4 (27)	51.9 (14/27)	1.34 (0.90, 1.99)	0.97 (0.67, 1.42)	
Use of LLINs					
No	5.0 (39)	53.8 (21/39)	1	1	0.5521
Yes	95.0 (746)	48.3 (360/746)	0.90 (0.66, 1.21)	0.93 (0.72, 1.19)	
No. of IPTp-SP doses					
0	22.7 (265)	58.9 (156/265)	1	1	< 0.0001
1	30.4 (239)	43.1 (103/239)	0.73 (0.61, 0.87)	0.67 (0.56, 0.80)	
2	20.2 (159)	44.0 (70/159)	0.75 (0.61, 0.92)	0.62 (0.50, 0.79)	
3+	15.8 (124)	41.9 (52/124)	0.71 (0.57, 0.90)	0.54 (0.41, 0.73)	

LLIN = long-lasting insecticidal net; PCR = polymerase chain reaction; IPTp-SP = intermittent preventive treatment of pregnant women with sulfadoxin pyrimethamine.

Prevalence was lower in women who had received SP than in women who had received no SP. In the adjusted model, prevalence was lower in women who had received SP than in women who had not received SP by 32% (95% CI, 20%–44%) after one dose, by 38% (95% CI, 21%–50%) in women who had received two doses, and by 46% (95% CI, 27%–59%) in women who had received three or more doses, and prevalence was higher in the second and third trimesters than in the first trimester.

## DISCUSSION

Malaria in pregnancy is a public health challenge that continues to put pregnant women and their unborn babies at risk. Our study found that asymptomatic carriage of *P. falciparum* infections not detected by RDT were common in pregnant women during the malaria transmission season in southern Senegal. Low-density infections can make an important contribution to malaria transmission,^16^ pregnant women represent an important potential reservoir in southern Senegal, and our findings indicate that the use of RDTs is not effective in detecting these infections as part of malaria elimination strategies. There is conflicting evidence as to whether low-density infections (submicroscopic or undetectable by RDT) are associated with adverse pregnancy outcomes, with some studies showing an association with anemia^17^^,^^18^ and low birth weight^19^^,^^20^ but others showing no evidence.^21^ The high prevalence of such infections in Senegal warrants further investigation of their clinical consequences. Our study shows that among asymptomatic women, RDTs had very low sensitivity. This contrasts with the study of Williams et al.,^22^ which found that a combination RDT (detecting HRPs and *Plasmodium* lactate dehydrogenase [pLDH]) had much higher sensitivity for detecting PCR-positive infections at the first antenatal visit in The Gambia, Burkina Faso, Mali, and Ghana. This study showed that RDTs were effective for malaria diagnosis in symptomatic patients, with a sensitivity of 98% and 83% for women with fever during unscheduled visits and scheduled visits, respectively. We were not able to investigate reasons for low RDT sensitivity in asymptomatic women, but this might be explained by low parasite densities and/or pfHRP2 gene deletion.^23^

The present study showed that almost half of pregnant women attending antenatal clinics were infected with *P. falciparum*. This high prevalence reflects the high level of malaria transmission in these areas during the rainy season. High prevalences have been reported in other studies.^22^^,^^24^^–^^26^ Our study confirmed the uneven distribution of malaria in southern Senegal, with a high prevalence in three regions—Kolda, Tambacounda, and Kedougou—compared with that in Kaffrine and Ndoffane. There has been a marked reduction in the number of reported cases of confirmed malaria in the north and central regions of Senegal over the last 15 years, associated with scaling up of control measures,^10^ but transmission remains high in the southern regions. Rainfall in Senegal follows an increasing north-south gradient, from 300 mm in the north to 1,200 mm in the south, with variations from 1 year to another.^27^ Consequently, conditions in the south of Senegal remain very favorable for the development of breeding sites for the vectors responsible for malaria transmission. The humidity following the rainfall also favors the longevity of the vector.^28^

We observed that parasite prevalence of infection determined by PCR was higher among women under 25 years old than among older women. Many studies have shown that high prevalences are found among young mothers.^25^^,^^29^ Adolescent girls are not a target group for NMCP strategies, which could put them at greater risk of disease.

Even if the prevalence is higher in primigravidae, we did not see an association of prevalence with gravidity that was observed in other studies.^30^ We found an association between gestational age and parasite prevalence. This association has been found in other studies.^25^

Women who had received SP were less likely to be PCR positive, with a 46% reduction in prevalence in women who had received three or more SP doses compared with women who had not received SP, in the adjusted model.^31^ Intake of SP was associated with marked reductions in prevalence consistent with effectiveness of IPTp in other studies.^25^^,^^31^^–^^34^ However, a study undertaken by the NMCP in Senegal indicates that these benefits may be poorly understood by women and by the heads of households,^35^ as women who reported having received IPTp-SP were unaware of the benefits. Better communication of the benefits of IPTp could lead to improved demand and uptake.

Use of LLINs was not associated with reduced prevalence of infection, in contrast to what other studies have shown. A limitation of our study is that LLIN use was self-reported by the participants, and it is possible that LLIN use was overreported.

As the RDTs are not suitable for the diagnosis of asymptomatic malaria, other strategies must be implemented to control the reservoirs of parasites that contribute to sustaining malaria transmission. In addition to measures to improve timely uptake of IPTp, there is also a need to protect primigravidae and to introduce systematic malaria screening during ANC.
